# There Is Selective Increase in Pro-thrombotic Circulating Extracellular Vesicles in Acute Ischemic Stroke and Transient Ischemic Attack: A Study of Patients From the Middle East and Southeast Asia

**DOI:** 10.3389/fneur.2019.00251

**Published:** 2019-03-19

**Authors:** Abdelali Agouni, Aijaz S. Parray, Naveed Akhtar, Fayaz A. Mir, Paula J. Bourke, Sujata Joseph, Deborah M. Morgan, Mark D. Santos, Muhammad F. Wadiwala, Saadat Kamran, Siveen K. Sivaraman, Ashfaq Shuaib

**Affiliations:** ^1^Department of Pharmaceutical Sciences, College of Pharmacy, Qatar University, Doha, Qatar; ^2^The Stroke Program, The Neuroscience Institute, Academic Health System, Hamad Medical Corporation, Doha, Qatar; ^3^Interim Translational Institute, Academic Health System, Hamad Medical Corporation, Doha, Qatar; ^4^Department of Medicine (Neurology), University of Alberta, Edmonton, AB, Canada

**Keywords:** extracellular vesicles (EVs), acute ischemic stroke (AIS), transient ischemic attacks (TIA), thrombosis, biomarkers

## Abstract

Stroke attacks were found to be present at a younger age in patients from Southeast Asia (SE) and the Middle East (ME) resident in the state of Qatar. Extracellular vesicles (EVs), which are small membrane vesicles with pro-thrombotic properties, may contribute to the high risk of stroke in this population. Thus, total and cell-specific medium size EVs were counted by flow cytometry in platelet-free plasma from healthy volunteers and patients with transient ischemic attacks (TIA) and acute ischemic stroke (AIS) from SE and ME. Acutely, within 48 h of attacks, there was an increase in total endothelial EVs in TIA (6.73 ± 1.77; *P* = 0.0156; *n* = 21) and AIS (11.23 ± 1.95; *P* = 0.0007; *n* = 66) patients compared to controls (2.04 ± 0.78; *n* = 24). Similar increases were also evident in EVs originating from platelets, erythrocytes, granulocytes, and leukocytes. Compared to controls, there was also an increase in EVs derived from activated endothelial cells, platelets, granulocytes, leukocytes, and pro-coagulant EVs (Annexin V^+^) at 5 and 30-days following the acute events, while a decrease was observed in erythrocyte-derived EVs. This is the first study characterizing EVs in TIA and AIS patients from ME and SE showing an increase in EVs associated with endothelial and platelet cell activation, which may contribute to the elevated risk of stroke at a younger age in this population.

## Introduction

Acute ischemic stroke (AIS) is the leading cause of disability and the second most common cause of death and dementia worldwide. The incidence of AIS is particularly high in Southeast Asia (SE) and the Middle East (ME) where the prevalence of hypertension and diabetes is high and an increasing proportion of the population are obese and suffer from metabolic syndrome (1–3).

Enhanced platelet aggregation and cell activation are important contributors to the pathophysiology and progression of AIS and are closely associated with stroke risk factors such as hypertension and diabetes. In addition, loss of vascular homeostasis and low-grade inflammation can further contribute to endothelial dysfunction and atherogenesis, a primary factor in the pathogenesis of AIS (4). In this context, circulating extracellular vesicles (EVs), particularly the endoplasmic membrane-derived microparticles or microvesicles, owing to their crucial pro-coagulant role may contribute to the onset and development of AIS (5).

EVs are membrane-containing vesicles that are released from activated or apoptotic cells of any type. EVs are classified into three main categories based on their size and their mechanism of cellular release: exosomes (30–130 nm), microparticles or microvesicles (100–1,000 nm), and the larger apoptotic bodies (>1,000 nm) (6). Based on the most recent position statement of the International Society for Extracellular Vesicles (ISEV), the term EVs was adopted throughout this article as a generic term to refer to lipid membrane-delineated, replication-defective, and not carrying a functional nucleus, naturally cell-shed vesicles (7). Elevated circulating levels of EVs, particularly those of medium size (microparticles), were reported in many cardiovascular diseases associated with inflammation and thrombotic alterations including stroke and coronary artery disease (8, 9). Total numbers of circulating EVs or those deriving from specific cell populations were found to correlate with the presence and severity of multiple disorders and are thus considered as biomarkers for the monitoring of disease progression. For instance, EVs originating from platelets were found to correlate with atypical carotid intima/media thickness in obese patients (10). In addition, high plasma numbers of pro-coagulant EVs were reported in patients suffering from metabolic syndrome (11, 12). Levels of EVs shed from endothelial cells (13) and platelets (14) were reported to increase in AIS. In another study, circulating EVs from platelets, endothelial cells, erythrocytes, leukocytes, lymphocytes, monocytes, and smooth muscle cells increased at the onset, and at 7 and 90 days in patients suspected with AIS compared to controls (15). Recently, however, Landers-Ramos et al. (16) reported that EVs levels of endothelial origin were not increased in chronic stroke patients compared to young and old healthy volunteers. Nonetheless, chronic stroke patients also suffering from diabetes had higher plasma levels of endothelial-derived EVs in comparison to those free of diabetes (16).

Studies that have investigated EVs in stroke were mostly conducted in small numbers of patients and in homogenous ethnic populations. There are currently no reports available of EVs in stroke patients from ME and SE. Furthermore, none of the available studies has investigated the levels of circulating EVs in patients with TIA, a major precursor and risk factor for AIS. Another crucial point, is that most previous studies only looked at the expression of select sub-populations of EVs in stroke patients.

The aim of the present study was therefore to count the number of circulating medium size EVs and phenotype them according to their cellular origins in patients with TIA and AIS patients from SE and ME origins resident the state of Qatar, at onset (within 48 h of attacks), 5 and 30-days following the initiation of appropriate pharmacotherapy.

## Materials and Methods

### Patients

The study was reviewed and approved by the Institutional Ethical Board (IRB) of Hamad Medical Corporation (#15304/15) and fully adhered to the principles of the declaration of Helsinki. All participants of the study gave written informed consent prior to their enrolment in the investigation. Patients were enrolled in the study within 48 h of stroke onset. Individuals aged 18 years and older and who gave written informed consent were included. Stroke was defined according to World Health Organization (WHO) criteria as “rapidly developing clinical signs of focal (or global) disturbance of cerebral function, with symptoms lasting 24 h or longer or leading to death, with no apparent cause other than vascular origin.” TIA was defined as a brief episode of neurologic dysfunction resulting from transient cerebral ischemia but that is not accompanied by cerebral infarction. All AIS and TIA patients received at Hamad General hospital were eligible for inclusion.

### Data Collection

The baseline medical history includes age, gender, ethnicity, nationality, marital status, household income, employment status, smoking status, alcohol intake, medical comorbidities (including a history of diabetes, dyslipidemia, coronary artery disease, peripheral vascular disease, chronic kidney disease, heart failure, and sleep apnea), and all current prescription and over-the-counter medications (including dosage, frequency and timing of administration). The cerebrovascular event type (AIS vs. TIA) was documented using the Trial of Org 10,172 in Acute Stroke Treatment (TOAST) classification and the date of stroke was recorded. This information is currently recorded routinely in the stroke database. We also recorded the type of work, duration of time since they moved to Qatar (for expatriate subjects) and the circumstances when the stroke occurred.

Physical examination included automated office blood pressure (BP) [taken with a BPTru® automated monitor by measuring six readings in the arm with the higher BP, discarding the first reading and averaging the latter two], height, weight, and waist circumference.

Laboratory tests included a complete blood count, electrolytes (sodium, potassium, chloride, total carbon dioxide), creatinine, fasting glucose, glycated hemoglobin (A1c), fasting lipid profile, c-reactive protein, and urine microalbumin. Computerized tomography (CT) head scan, echocardiogram, and standard (not 3D) carotid ultrasound results were performed as part of routine clinical care at the time of stroke were recorded in the case record forms (CRF). These tests are performed as part of routine clinical care.

### Isolation of Platelet-Free Plasma (PFP) From Whole Blood

Peripheral venous blood (10 mL) from control subjects, AIS, and TIA patients was obtained in ethylenediaminetetraacetic acid tubes (Vacutainers; Becton Dickinson) by using a 21-gauge needle to reduce platelet stimulation. Samples were assayed within 2 h of collection at room temperature. Blood samples were then subjected low speed centrifugation (270 × *g*) for 20 min to separate platelet-rich plasma from whole blood. Following this, platelet-rich plasma was centrifuged for further 20 min at a speed of 1,500 × *g* to collect PFP, which was then frozen and stored at −80°C until subsequent analysis for EVs content (11, 12, 17, 18).

### Count and Identification of Cellular Origins of EVs

Specific cellular subpopulations of medium size EVs were identified in PFP according the expression of surface-specific proteins as previously done by us (11, 12, 17, 18). Specific fluorescent antibodies targeting the following surface antigens were used in this study: PE-CD146 (endothelial cells), PE-CD41 (platelets), PC7-CD45 (leukocytes), PC7-CD235a (erythrocytes), PE-CD62E (E-Selectin^+^ EVs), and PE-CD62P (P-Selectin^+^ EVs) (Beckman Coulter through Sedeer Medical, Doha, Qatar). Irrelevant human IgG were used as isotype-matched negative controls for each sample (Beckman Coulter). To count medium size EVs and determine plasma concentrations for each cell origin, separate assays for each surface marker were conducted. Briefly, 10 μL of PFP from healthy controls or patients were incubated with 5 μL of each specific antibody for 45 min protected from light. Then, samples were suspended in 300 μL of 0.9% saline solution and 10 μL Flowcount microbeads (Beckman Coulter), a mix of fluorescent beads with known concentration used to calculate absolute counts, were mixed before samples were analyzed by flow cytometry using a BD LSRFortessa analyzer (BD Biosciences, USA) as previously shown by us (11, 12, 17, 18).

For the identification of the number of pro-coagulant EVs (expressing phosphatidylserine), separate assays were conducted to determine Annexin V binding. Briefly, 2 μL of Annexin V (Beckman Coulter) were incubated with 5 μL PFP from each participant for 30 min protected from light. Then, samples were suspended in 300 μL of Annexin-V labeling buffer, and 5 μL (equal volume to sample) of Flowcount microbeads (Beckman Coulter) were added before the analysis of samples by flow cytometry using a BD LSRFortessa analyzer (BD Biosciences) as previously shown by us (11, 12, 17, 18).

### Statistical Data Analysis

Data are expressed as mean ± SEM or mean ± SD; *n* indicates the number of subjects included in each experimental group. Statistical analyses were performed by Mann-Whitney *U* or analysis of one-way variance (ANOVA) for repeated measures and subsequent Tukey's *post-hoc* test. *P* < 0.05 was accepted as statistically significant. Analyses were performed using GraphPad Prism 7 software (GraphPad Software Inc., San Diego, USA).

## Results

### Patients Baseline Characteristics

Between September 2016 and May 2018, we recruited 24 healthy controls and a total of 119 patients with suspected acute stroke, of whom 87 patients (66 [76%] AIS and 21 [24%] TIA) were eventually included in the study after exclusion of stroke mimics.

The baseline characteristics of the 24 healthy volunteers and the 87 patients included in the study are shown in Table 1. Similar to previous experience, the average age in both TIA (48.6 ± 9.5) and AIS patients (50.5 ± 11.1) was young (3). The patient population was predominantly male in AIS patients (95.5%). In terms of disease classification, most AIS patients suffered from a small vessel disease (59.1%), followed by cardio-embolic attacks (16.7%) and large vessel disease (15.2%) (Table 1).

**Table 1:** Baseline Characteristics of subjects enrolled in the study.

	**Controls (*n =* 24)**	**Transient Ischemic Attack (*n =* 21)**	**Acute Ischemic Stroke (*n =* 66)**	***P* value**
Age (years)	47.7 ± 7.4	48.6 ± 9.5	50.5 ± 11.1	0.485
**GENDER**				
Male	18 (75)	17 (81.0)	63 (95.5)	0.033
Female	6 (25)	4 (19.0)	3 (4.5)	
**ETHNICITY**				
ME	6 (29)	12 (57.1)	22 (33.3)	0.013
SE	18 (71)	9 (42.9)	44 (66.7)	
Prior use of statins	0	7 (33.3)	8 (12.1)	0.025
Prior use of anti-hypertensive	0	6 (28.6)	12 (18.2)	0.306
Prior use of anti-diabetic medications	0	3 (14.3)	10 (15.2)	0.923
Diabetic on admission	0	7 (33.3)	27 (40.9)	0.535
Hypertensive on admission	0	13 (61.9)	45 (68.2)	0.595
Dyslipidemia on admission	0	15 (71.4)	38 (57.6)	0.257
Previous stroke	0	1 (4.8)	2 (3.0)	0.705
Coronary artery disease	0	2 (9.5)	6 (9.1)	0.952
Atrial fibrillation	0	1 (4.8)	3 (4.5)	0.967
Smoking history	0	6 (28.6)	29 (43.9)	0.211
*IV* thrombolysis received	0	2 (9.5)	12 (18.2)	0.347
**PROGNOSIS AT DISCHARGE**				
Good (mRS 0–2)		21 (100.0)	48 (72.7)	0.007
Poor (mRS 3–6)		0.0	18 (27.3)	
**PROGNOSIS AT 90-DAYS**				
Good (mRS 0–2)		20 (95.2)	57 (86.4)	0.267
Poor (mRS 3–6)		1 (4.8)	9 (13.6)	
**TOAST CLASSIFICATION**				
Small vessel disease			39 (59.1)	
Large vessel disease			10 (15.2)	
Cardio-embolic			11 (16.7)	
Stroke of determined origin			3 (4.5)	
Stroke of undetermined origin			3 (4.5)	
**OTHER**				
Small vessel disease present		11 (52.4)	39 (59.1)	0.588
Silent infarct present		4 (19.0)	25 (37.9)	0.111
White matter ischemia present		14 (66.7)	52 (78.0)	0.258
Cerebral micro-bleeds present		4 (19.0)	20 (30.3)	0.315
Admission NIHSS		1.67 ± 2.1	3.35 ± 3.4	0.035
Systolic blood pressure		162.4 ± 26.1	157.4 ± 33.1	0.522
Diastolic blood pressure		94.7 ± 17.1	93.9 ± 21.0	0.883
Body Mass Index		27.6 ± 3.1	27.1 ± 3.9	0.634
HbA1c levels		6.4 ± 1.4	6.7 ± 2.1	0.545
Cholesterol levels		4.9 ± 1.3	5.2 ± 1.2	0.557
Triglyceride levels		1.9 ± 1.0	1.6 ± 0.9	0.353
HDL levels		1.1 ± 0.7	0.9 ± 0.2	0.031
LDL levels		3.1 ± 1.0	3.5 ± 1.0	0.086

*Results are presented as mean ± SD or number (%) as appropriate. P-value from one-way ANOVA for quantitative variables and from Chi-square analysis for qualitative variables*.

*mRS, modified Rankin scale; NIHSS, national institutes of health stroke scale*.

### Total Number of Circulating of EVs and Their Cellular Origins at Onset of Attacks

**Figure 1:**
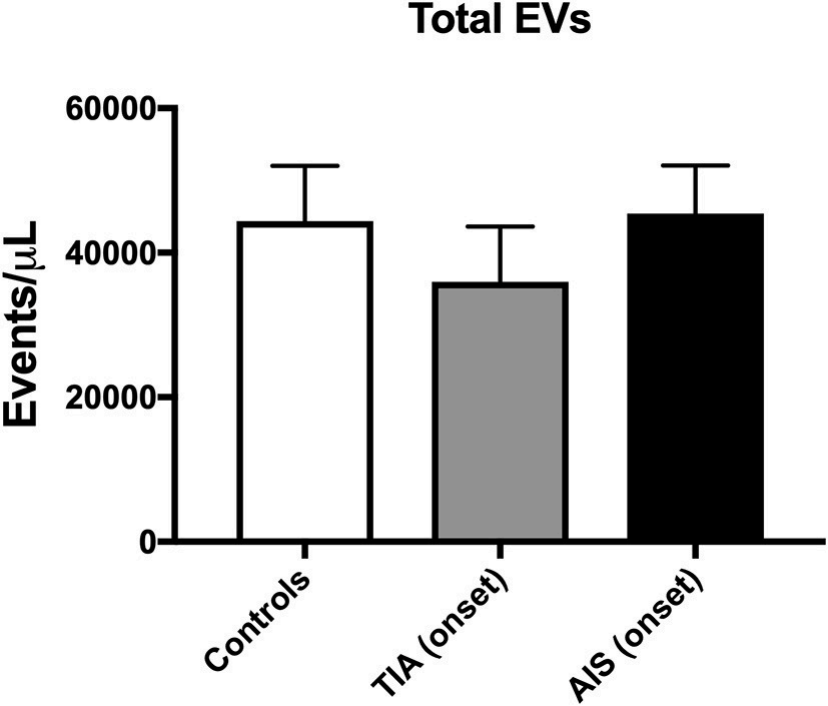
Circulating total EV levels, at onset of attacks, in patients with TIA and AIS compared to healthy controls. Histograms represent events/μL in plasma poor in platelets (PFP) expressed as mean ± SEM. Controls, *n* = 24; TIA, *n* = 21; AIS, *n* = 66.

**Figure 2:**
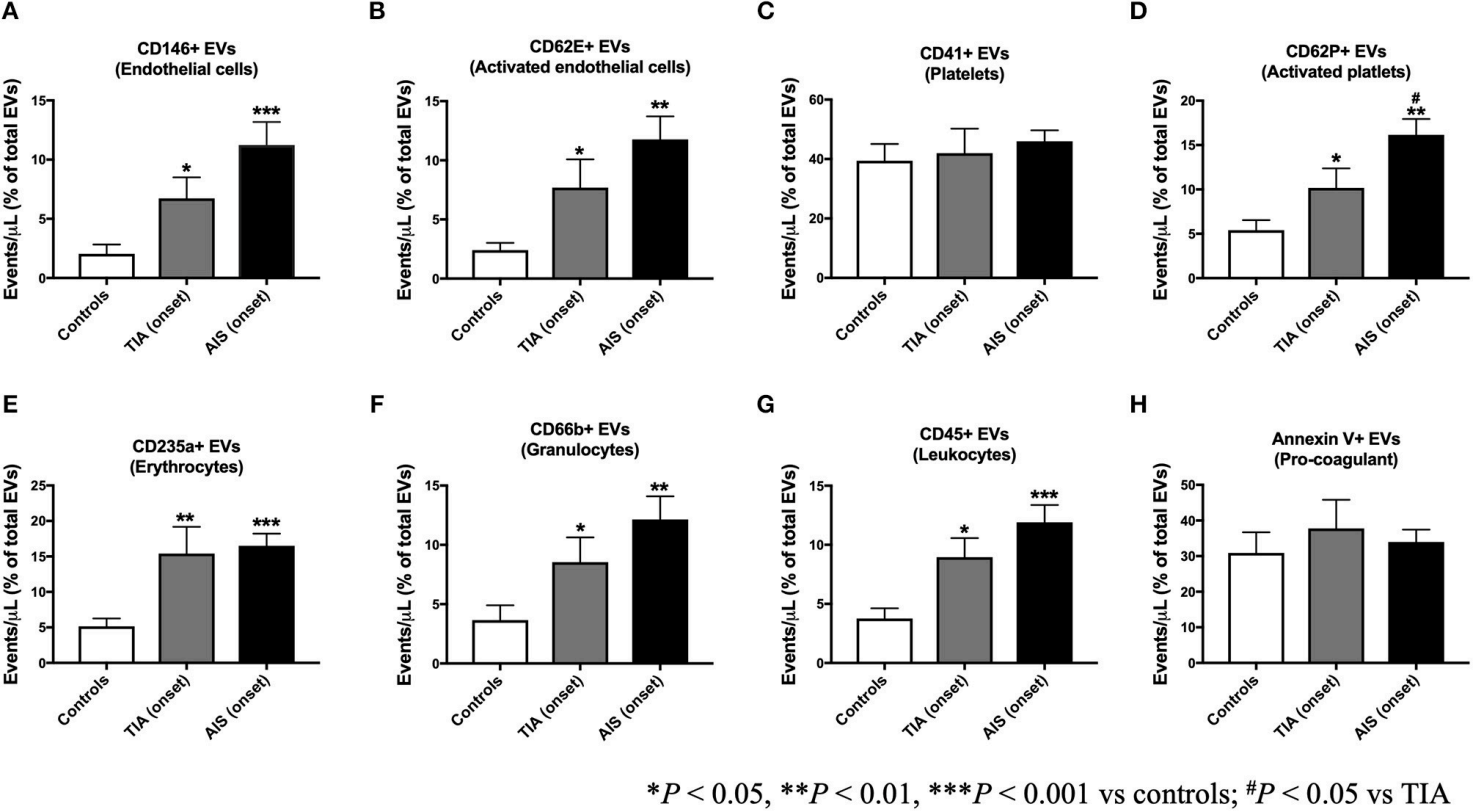
Circulating EV levels, expressed as percentage of total EVs, at onset of attacks, in patients with TIA and AIS compared to healthy controls. Histograms represent circulating levels of EVs derived from: **(A)** endothelial cells (CD146^+^), **(B)** activated endothelial cells (CD62E^+^), **(C)** platelets (CD41), **(D)** activated platelets (CD62P^+^), **(E)** erythrocytes (CD235a^+^), **(F)** granulocytes (CD66b^+^), **(G)** leukocytes (CD45^+^), **(H)** and pro-coagulant (Annexin V^+^) origins. Histograms represent events/μL (% of total EVs) in PFP expressed as mean ± SEM. Controls, *n* = 24; TIA, *n* = 21; AIS, *n* = 66. *P < 0.05, **P < 0.01, ***P < 0.001 vs. controls; ^#^*P* < 0.05 vs. TIA.

The count of total EVs regardless of their cellular origin did not differ between controls, TIA and AIS patients (Figure 1) within 48 h of onset of attacks. TIA and AIS patients had significantly higher percentage of circulating EVs derived from endothelial cells (CD146^+^; Figure 2A) and activated endothelial cells that express E-selectin (CD62E^+^; Figure 2B) compared to healthy volunteers. Furthermore, there was a trend for AIS patients to express more of total and activated endothelial-derived EVs compared to TIA patients (Figures 2A,B).

Whereas, platelet-derived EVs (CD41^+^) were not significantly different between controls, TIA, or AIS patients (Figure 2C), the number of EVs derived from activated platelets expressing P-selectin (CD62P^+^) was significantly higher in TIA and AIS patients compared to controls (Figure 2D). Interestingly, AIS patients exhibited a significantly higher percentage of activated platelets-derived EVs compared to TIA patients (Figure 2D).

EVs derived from erythrocytes (CD235a^+^) were found to be significantly higher in the blood from both TIA and AIS patients compared to healthy volunteers (Figure 2E). Similarly, EVs derived from circulating immune cells, granulocytes (CD66^+^; Figure 2F) and leukocytes (CD45^+^; Figure 2G), were also significantly higher in TIA and AIS patients compared to controls although there was a non-significant trend for AIS patients to express more of these two EV subtypes compared to TIA patients (Figures 2F,G). Finally, the percentage of pro-coagulant EVs expressing Annexin V at their surface was not significantly different between the three groups within 48 h of onset of attacks (Figure 2H).

### The Total Number of EVs Increased Over Time in AIS and TIA Patients as a Percentage of Onset Levels

**Figure 3:**
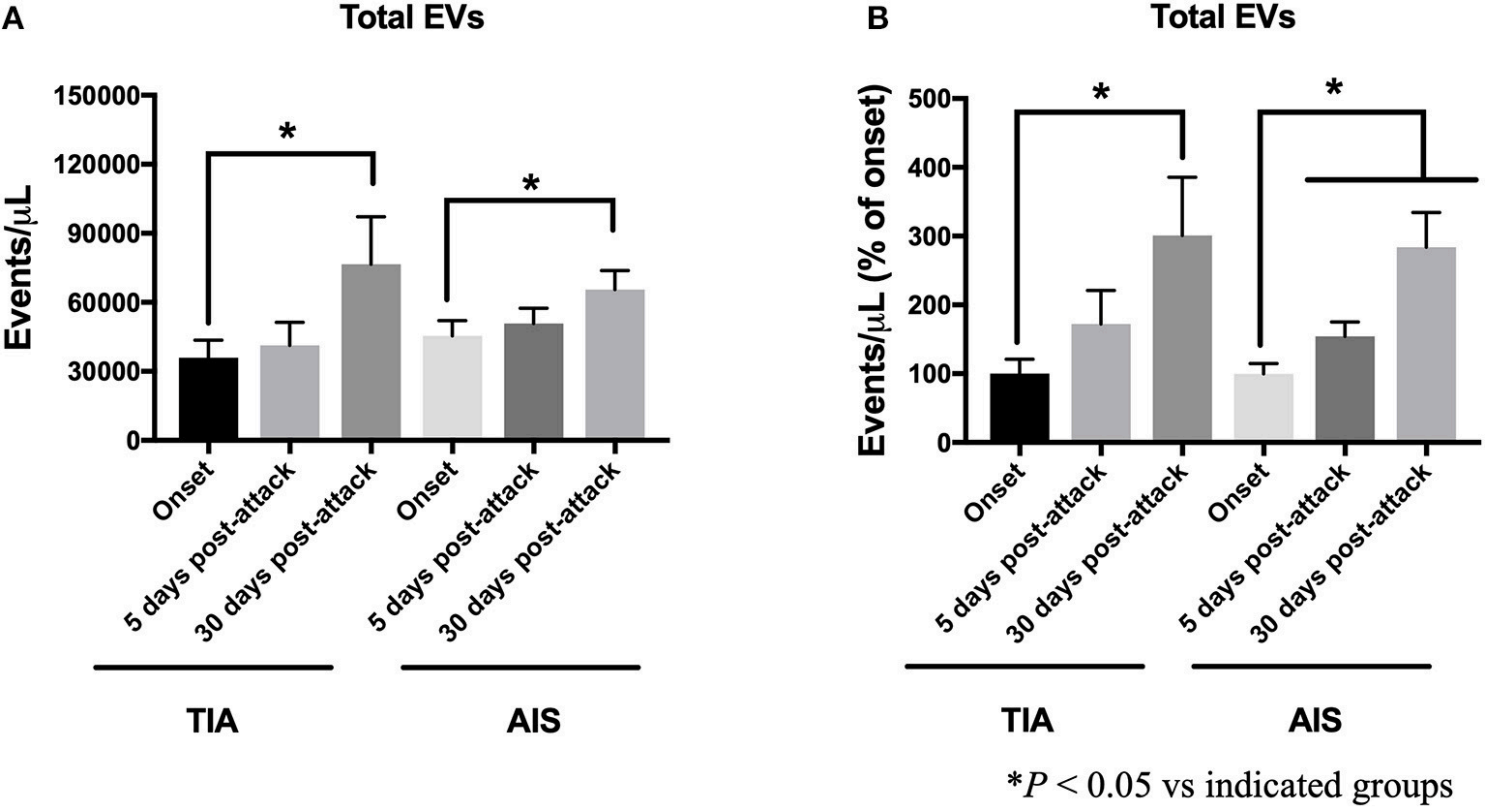
Circulating total EV levels, at onset, 5-days and 30-days post-attacks in patients with TIA and AIS patients. **(A)** Histograms represent total EVs expressed as events/μL in PFP. **(B)** Histograms represent total EVs expressed as events/μL (% of onset levels) in PFP. Data are expressed as mean ± SEM. Controls, *n* = 24; TIA, *n* = 21; AIS, *n* = 66. *P < 0.05 vs. indicated groups.

To investigate the impact of disease progression and the initiation of treatment on the expression of EVs, we assessed the progression of numbers of total EVs over time at 5- and 30-days post-attacks (Figure 3). The counts of EVs are expressed either as absolute numbers or as percentage of increase compared to the levels observed at onset of attacks for every patient. As shown in Figure 3A, the absolute number of total EVs continued to increase over time in both TIA and AIS patients, with the levels at the 30-days' time point being the most significant increase. As observed in Figure 3B, the number of total EVs, expressed as percentage of onset levels, also steadily increased over time at 5- and 30-days post-attacks both in TIA and AIS patients.

### EVs From Selected Cellular Origins Increased Over Time Compared to Onset Levels

**Figure 4:**
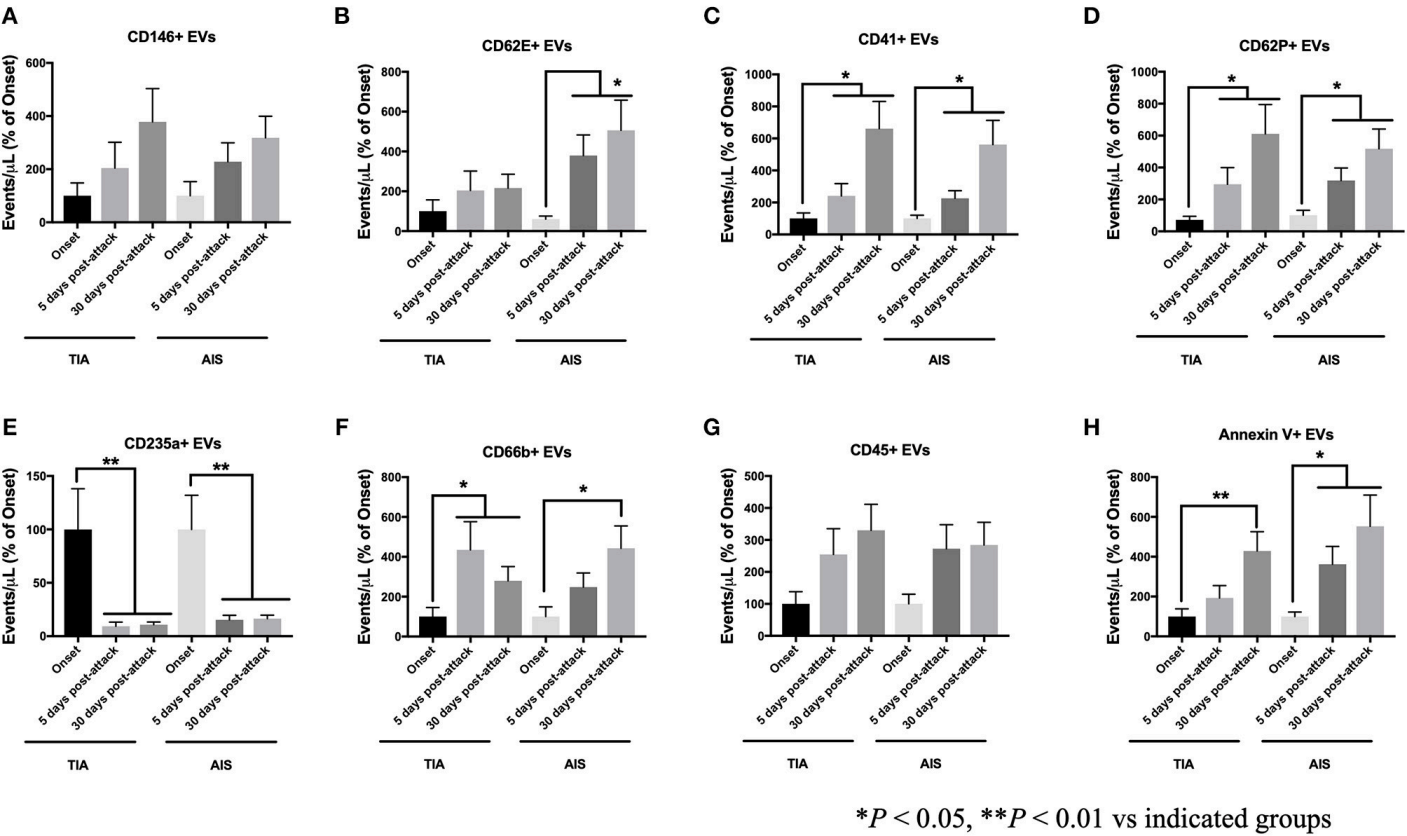
Circulating EV levels, expressed as events/μL (% of onset levels), at onset, 5-days and 30-days post attacks, in PFP from patients with TIA and AIS. Histograms represent circulating levels of EVs derived from: **(A)** endothelial cells (CD146^+^), **(B)** activated endothelial cells (CD62E^+^), **(C)** platelets (CD41), **(D)** activated platelets (CD62P^+^), **(E)** erythrocytes (CD235a^+^), **(F)** granulocytes (CD66b^+^), **(G)** leukocytes (CD45^+^), **(H)** and pro-coagulant (Annexin V^+^) origins. Data are expressed as mean ± SEM. Controls, *n* = 24; TIA, *n* = 21; AIS, *n* = 66. *P < 0.05, **P < 0.01 vs. indicated groups.

The increase in EVs at 5- and 30-days post-attacks varied depending on their cells of origin. TIA and AIS patients had higher levels of EVs derived from endothelial (CD146^+^; Figure 4A) and activated endothelial cells (CD62E^+^; Figure 4B) at 5- and 30-days compared to onset levels. The increase in EVs from activated endothelial cells (CD62E^+^; Figure 4B) was highest in AIS patients, indicating a stronger activation of endothelial cells as the disease progresses in AIS patients compared to TIA patients. Similarly, EVs derived from both platelets (CD41^+^; Figure 4C) and activated platelets (CD62P^+^; Figure 4D) also significantly increased over time at 5- and 30-days in both TIA and AIS patients, indicating a sustained activation of platelets despite appropriate treatment being started. The expression of EVs derived from immune cells, granulocytes (CD66b^+^; Figure 4F) and leukocytes (CD45^+^; Figure 4G), also increased over time in both TIA and AIS patients, indicating a sustained activation of these cell types on the short-term following the start of pharmacotherapy. However, the levels of erythrocyte-derived EVs (CD235a^+^; Figure 4E) decreased significantly over time at 5- and 30-days post-attacks in both TIA and AIS patients, suggesting a reduction in the activation of these cells over time following the initiation of therapy.

Although at onset of attacks, circulating levels of pro-coagulant EVs (Annexin V^+^) were not different between controls, TIA and AIS (Figure 2), the numbers of Annexin V^+^ EVs, expressed as a proportion of onset levels, increased with time in both TIA and AIS patients at 5- and 30-days post-attacks (Figure 4H), indicating that the shedding of pro-coagulant EVs increased over time.

### As a Proportion of Total EVs at Each Time Point, the Initiation of Treatment Did Not Affect the Short-Term Expression of EVs Both in TIA and AIS Patients

**Figure 5:**
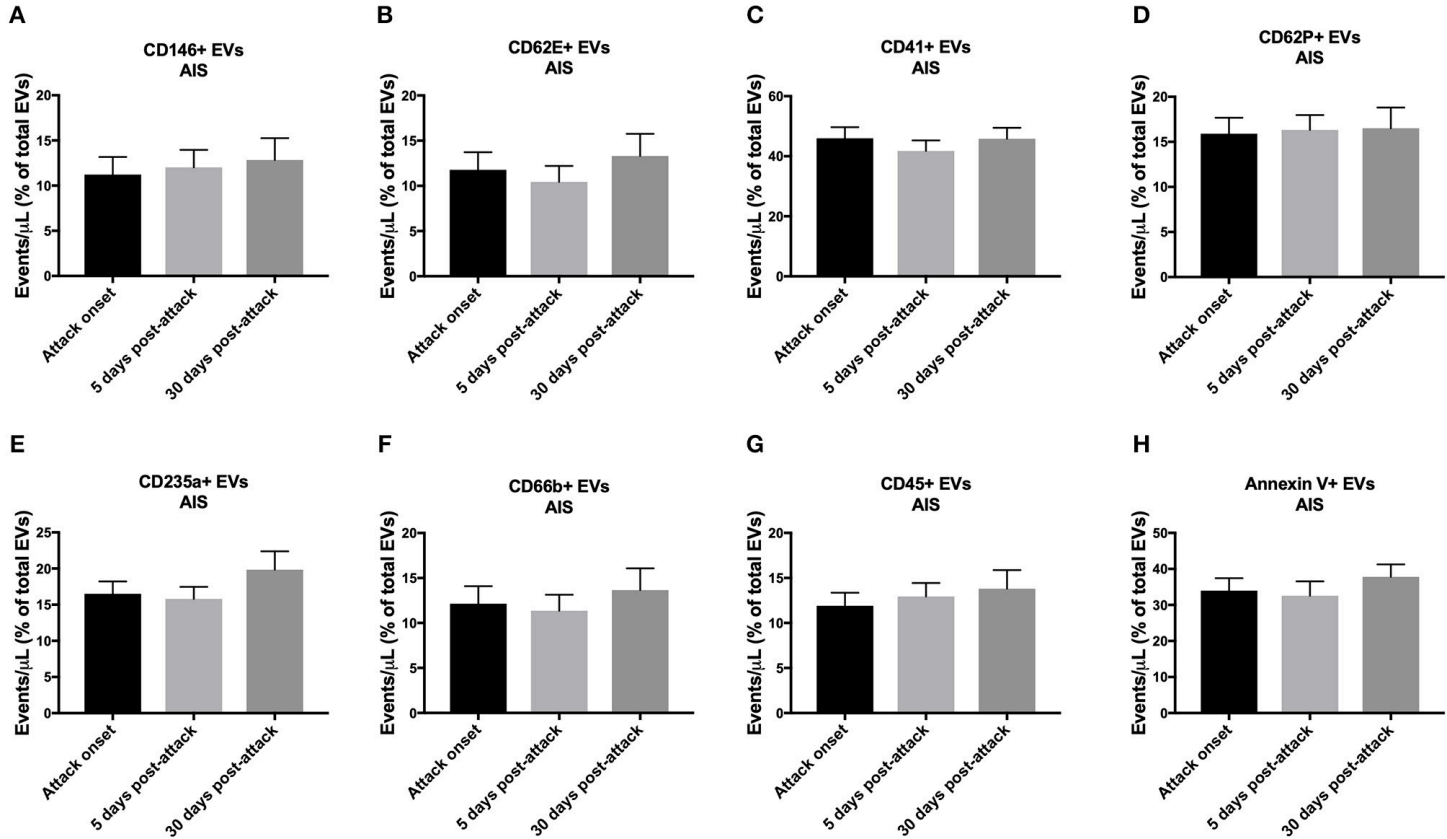
Circulating EV levels, expressed as events/μL (% of total EVs), at onset, 5-days and 30-days post-attacks in PFP from patients with AIS. Histograms represent circulating levels of EVs derived from: **(A)** endothelial cells (CD146^+^), **(B)** activated endothelial cells (CD62E^+^), **(C)** platelets (CD41), **(D)** activated platelets (CD62P^+^), **(E)** erythrocytes (CD235a^+^), **(F)** granulocytes (CD66b^+^), **(G)** leukocytes (CD45^+^), **(H)** and pro-coagulant (Annexin V^+^) origins. Data are expressed as mean ± SEM. AIS, *n* = 66.

**Figure 6:**
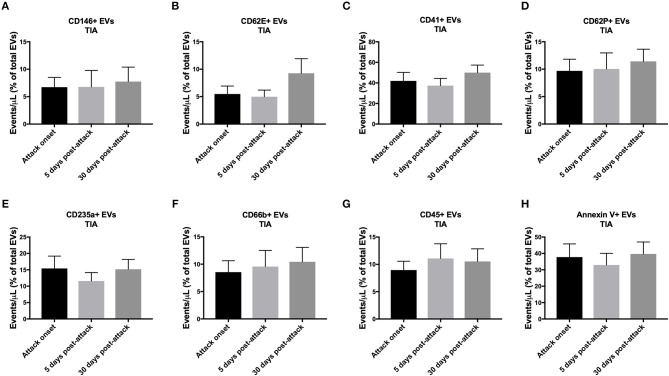
Circulating EV levels, expressed as events/μL (% of total EVs), at onset, 5-days and 30-days post-attacks in PFP from patients with TIA. Histograms represent circulating levels of EVs derived from: **(A)** endothelial cells (CD146^+^), **(B)** activated endothelial cells (CD62E^+^), **(C)** platelets (CD41), **(D)** activated platelets (CD62P^+^), **(E)** erythrocytes (CD235a^+^), **(F)** granulocytes (CD66b^+^), **(G)** leukocytes (CD45^+^), **(H)** and pro-coagulant (Annexin V^+^) origins. Data are expressed as mean ± SEM. TIA, *n* = 21.

As shown in Figure 5, the levels of circulating EVs, expressed as a percentage of total EVs at each time point, from all the cell origins investigated did not change in AIS patients over the monitored period (5- and 30-days post-attacks). Similar pattern was observed with TIA patients, where circulating levels of EVs from all cell origins studied did not vary, as a percentage of total EVs for each time point, after 5- and 30-days following the initiation of the pharmacotherapy (Figure 6). These data demonstrate that, as a percentage of total EVs at each time point, circulating levels of EVs from various cellular origins were constant over time (at 5- and 30-days) although their numbers have increased compared to onset time point. The increased number of total EVs over time drives the increase of specific populations of EVs without affecting their overall proportion in blood. Together, these findings further indicate that in both TIA and AIS patients, cellular activation continued to be elevated on the short-term despite the management of risk factors and the administration of appropriate pharmacotherapy to the patients.

## Discussion

For many decades, EVs were considered as inert cell debris or platelet dust derived from platelets which are rich in phospholipids and endowed of pro-coagulant capacity (8). Later, it was found that EVs could transport cargo content including secretable and non-secretable biological molecules such as active lipids, nucleic acids (microRNA and mRNA) in addition to membrane and cytosolic proteins to target cells (8, 19, 20). Circulating EVs derive from platelets, red and white blood cells, endothelial cells, and monocytes; however, most EVs found in the blood are from platelet origin (8, 9). EVs regulate inflammation and have pro-coagulant properties (21). Plasma numbers of EVs are elevated in cardiovascular disorders associated with thrombotic alterations including stroke and coronary artery disease (8, 9). Plasma levels of total EVs or those deriving from specific cell populations were found to correlate with the presence and severity of multiple disorders and are thus considered as biomarkers for the monitoring of disease progression and hence can provide a signature for changes occurring inside the body (11, 12, 22).

We report in the current study that although the total number of EVs did not differ between TIA and AIS patients compared to controls within 48 h of attacks onset, both TIA and AIS patients had higher circulating levels of EVs derived from endothelial cells (CD146^+^), activated endothelial cells (CD62E^+^), activated platelets (CD62P^+^), granulocytes (CD66b^+^), and leukocytes (CD45^+^) compared to controls; however, AIS patients had higher numbers compared to TIA patients. While there are few reports regarding the phenotype of expression of circulating EVs in AIS, there is a severe paucity of information regarding EVs numbers in blood from TIA patients and how they differ compared to AIS patients. To the best of our knowledge, this is the first report to determine circulating levels of EVs from various cell origins in TIA patients and compare them to AIS patients and healthy controls. Only one previous publication could be found reporting increased levels of platelet-derived EVs in TIA compared to controls (23).

In relation to AIS, our findings are partly in agreement with the few previous studies which investigated EVs levels in AIS patients. Li et al. (13) have studied EVs in a cohort composed of patients with AIS and gender-matched healthy volunteers. In accordance with our data, authors reported that patients had higher levels of endothelial-derived EVs and EVs carrying markers of cell activation (CD62E^+^); however, no differences in platelet-derived EVs were noted (13). Another study, reported in a cohort of AIS patients that platelet-derived EVs were, however, increased compared to control subjects (14). Recently, it was observed that plasma levels of endothelial-derived EVs were not increased in chronic stroke patients compared to younger and old healthy volunteers (16). These findings are in contrast with our observations and the discrepancy may be attributable to the ethnic differences between the study cohorts [Chinese in Chen et al. (14) vs. SE and ME in our study]. In addition, the study by Chen et al. (14), determined the levels of platelet-derived EVs using anti-CD61 antibody, while in our study we have used an anti-CD41 antibody. Both CD41 and CD61 are platelet-specific glycoproteins, which were widely used as surface markers for the detection of EVs from platelet origin (24, 25). However, in our hands, we have observed similar results to those observed with CD41 when CD61 was used as a surface marker to detect platelet-derived EVs (data not shown for CD61) and hence the discrepancy between the **two** studies may be more related to the ethnic differences in study populations.

More recently, circulating EVs from platelets, endothelial cells, erythrocytes, leukocytes, lymphocytes, monocytes, and smooth muscle cells were analyzed in patients with suspected AIS at the onset of attacks, and then at 7 and 90 days. In line with our observations reported here, authors observed that EVs from all these cell origins were increased in stroke patients compared to controls at onset of attacks and that their levels remained high at 7 and 90 days following the acute events (15). In our hands, we observed that, by contrast to Chiva-Blanch et al. (15), the number of EVs from endothelial cells, activated endothelial cells, platelets, activated platelets, granulocytes, leukocytes, and pro-coagulant origin, expressed as percentage of onset levels, all increased in both TIA and AIS patients after 5 and 30 days post-attacks and the initiation of treatment and the management of risk factors (e.g., diabetes or hypertension). These data, show an escalated cell activation, particularly in endothelial cells and platelets, acutely after the attacks in both TIA and AIS patients, which may contribute to heightened endothelial and pro-thrombotic activities contributing thus to an elevated risk of stroke recurrence. However, we cannot rule out that the elevation of EVs levels following the attacks and the start of pharmacotherapy might be related to the resolution of thrombi and the secondary consequences of ischemic episodes. Thus, a longer follow-up of these patients is warranted to determine the long-term changes of EVs and further ascertain the prognostic value of circulating EVs levels in TIA and AIS patients with regards to the risk of recurrence.

Of particular interest, in our study, plasma levels of EVs from platelets (CD41^+^), activated platelets (CD62P^+^), and pro-coagulant (Annexin V^+^) origins increased, as a percentage of onset numbers, on the short-term (at 5- and 30-days after acute attacks), indicating a higher number of EVs with pro-thrombotic activity, which may contribute to the increased risk of recurrence of stroke in these patients, including those with no major consequences (TIA). EVs from platelet origin are indeed strongly pro-thrombotic (21). It has been reported that one single platelet-derived EV had nearly the same pro-coagulant activity as one activated platelet despite the surface area was 2 orders of magnitude smaller, suggesting that the surface of platelet-derived EVs is 50 to 100–fold more pro-coagulant than that of activated platelets (26). Furthermore, though not directly assessed in this study, EVs expressing tissue factor (TF) may also enhance the coagulation process in stroke patients. EVs carrying TF were found to be highly expressed in several ischemic conditions such as atherosclerosis and acute coronary syndromes (27). It was reported that the expression of TF at the surface of EVs enhanced their pro-coagulant response (28). Higher circulating levels of EVs expressing TF were observed in AIS patients compared to healthy controls. Moreover, 1 week after the diagnosis, the activity of EVs was found to be more elevated in stroke patients not treated with tissue plasminogen activator compared to their basal activity at the onset of attacks (29).

While we have observed that erythrocyte-derided EVs (CD235a^+^) were increased to the same extent in both TIA and AIS compared to controls within 48 h of onset of attacks, we found that their levels, expressed as a proportion of onset levels, decreased in both TIA and AIS patients at 5- and 30-days following the initiation of pharmacotherapy. In contrast with our observation, Chiva-Blanch et al. (15), found that erythrocyte-derived EVs were higher in AIS patients compared to controls and continued to be high at 7 and 90 days post-stroke attacks (15). Our data suggest that the administration of treatment to patients led to a decrease in the expression of erythrocyte-derided EVs, which were previously linked to hypercoagulable states. Consistent with our observations, Tan et al. (30), observed that high circulating levels of phosphatidylserine-positive erythrocyte-derived EVs, which were highly pro-coagulant, in patients suffering from the hypercoagulable status of polycythemia vera and that the treatment of patients with hydroxyurea was associated with a decrease in the shedding of EVs from erythrocytes (30).

Our data characterized for the **first** time circulating EVs in patients with AIS and compared them to TIA in a multiethnic population from SE and ME origins. We provided here, a very comprehensive analysis of most circulating EVs from vascular wall, blood, and immune cells. We found here that EVs of various origins, especially those associated with endothelial cell injury and platelet activation, are increased in TIA and AIS patients and that their levels persist to be high on the short-term up to 30-days post-attacks, indicating a sustained cellular activation, which may be associated with a heightened risk of recurrence of acute events. Therefore, longer follow-up studies are required to ascertain the value of EVs as biomarkers for the risk of stroke recurrence, especially in a population presenting with stroke at young age like this one.

## Data Availability

The datasets generated for this study are available on request to the corresponding author.

## Author Contributions

AA and AS wrote the article and all authors contributed to the drafting of the manuscript and its critical revision for intellectual content. AA, AS, and ASP were involved in the study conception and design and in the interpretation of data. AA and AS acquired funding and supervised the experiments. AA, ASP, FM, and SS conducted sample analysis, and data acquisition, processing, statistical analysis, and interpretation. AS oversaw the whole clinical process and contributed to the analysis and interpretation of data. NA, PB, SJ, DM, MS, MW, and SK collected clinical data, refined diagnosis, and collected clinical samples for the study. NA contributed to the interpretation of clinical data.

### Conflict of Interest Statement

The authors declare that the research was conducted in the absence of any commercial or financial relationships that could be construed as a potential conflict of interest.

## References

[1] Brainin M Teuschl Y Kalra L. Acute treatment and long-term management of stroke in developing countries. Lancet Neurol. (2007) 6:553–61. 10.1016/S1474-4422(07)70005-4 17509490

[2] Feigin VL Roth GA Naghavi M Parmar P Krishnamurthi R Chugh S. Global burden of stroke and risk factors in 188 countries, during 1990–2013: a systematic analysis for the Global Burden of Disease Study 2013. Lancet Neurol. (2016) 15:913–24. 10.1016/S1474-4422(16)30073-4 27291521

[3] Akhtar N Salam A Kamran S D'Souza A Imam Y Own A Pre-existing Small vessel disease in patients with acute stroke from the Middle East, Southeast Asia, and Philippines. Transl Stroke Res. (2018) 9:274–82. 10.1007/s12975-017-0578-7 29101611

[4] Jensen HA Mehta JL. Endothelial cell dysfunction as a novel therapeutic target in atherosclerosis. Expert Rev Cardiovasc Ther. (2016) 14:1021–33. 10.1080/14779072.2016.1207527 27362558

[5] Wang B Cai W Zhang Z Zhang H Tang K Zhang Q. Circulating microparticles in patients after ischemic stroke: a systematic review and meta-analysis. Rev Neurosci. (2018). [Epub of print]. 10.1515/revneuro-2017-0105. 29750657

[6] Chiang CY Chen C. Toward characterizing extracellular vesicles at a single-particle level. J Biomed Sci. (2019) 26:9. 10.1186/s12929-019-0502-4 30646920 PMC6332877

[7] Thery C Witwer KW Aikawa E Alcaraz MJ Anderson JD Andriantsitohaina R. Minimal information for studies of extracellular vesicles 2018 (MISEV2018): a position statement of the International Society for Extracellular Vesicles and update of the MISEV2014 guidelines. J Extracell Vesicles. (2018) 7:1535750. 10.1080/20013078.2018.1535750 30637094 PMC6322352

[8] Agouni A Andriantsitohaina R Martinez MC. Microparticles as biomarkers of vascular dysfunction in metabolic syndrome and its individual components. Curr Vasc Pharmacol. (2014) 12:483–92. 10.2174/1570161112666140423223148 24846237

[9] Chen Y Li G Liu ML. Microvesicles as Emerging biomarkers and therapeutic targets in cardiometabolic diseases. Genomics Proteomics Bioinformatics. (2018) 16:50–62. 10.1016/j.gpb.2017.03.006 29462670 PMC6000161

[10] Csongradi E Nagy B Jr Fulop T Varga Z Karanyi Z Magyar MT. Increased levels of platelet activation markers are positively associated with carotid wall thickness and other atherosclerotic risk factors in obese patients. Thromb Haemost. (2011) 106:683–92. 10.1160/TH11-01-0030 21866298

[11] Agouni A Lagrue-Lak-Hal AH Ducluzeau PH Mostefai HA Draunet-Busson C Leftheriotis G. Endothelial dysfunction caused by circulating microparticles from patients with metabolic syndrome. Am J Pathol. (2008) 173:1210–9. 10.2353/ajpath.2008.080228 18772329 PMC2543087

[12] Agouni A Ducluzeau PH Benameur T Faure S Sladkova M Duluc L. Microparticles from patients with metabolic syndrome induce vascular hypo-reactivity via Fas/Fas-ligand pathway in mice. PLoS ONE. (2011) 6:e27809. 10.1371/journal.pone.0027809 22110764 PMC3217000

[13] Li P Qin C. Elevated circulating VE-cadherin^+^CD144^+^endothelial microparticles in ischemic cerebrovascular disease. Thromb Res. (2015) 135:375–81. 10.1016/j.thromres.2014.12.006 25523345

[14] Chen Y Xiao Y Lin Z Xiao X He C Bihl JC. The role of circulating platelets microparticles and platelet parameters in acute ischemic stroke patients. J Stroke Cerebrovasc Dis. (2015) 24:2313–20. 10.1016/j.jstrokecerebrovasdis.2015.06.018 26169549 PMC4592794

[15] Chiva-Blanch G Suades R Crespo J Pena E Padro T Jimenez-Xarrie E. Microparticle shedding from neural progenitor cells and vascular compartment cells is increased in ischemic stroke. PLoS ONE. (2016) 11:e0148176. 10.1371/journal.pone.0148176 26815842 PMC4729528

[16] Landers-Ramos RQ Serra MC Blumenthal JB Ryan AS Hafer-Macko CE Prior SJ. Type 2 diabetes and older age contribute to elevated plasma microparticle concentrations independent of chronic stroke. Exp. Physiol. (2018) 103:1560–70. 10.1113/EP087116 30062787 PMC6449859

[17] Mostefai HA Meziani F Mastronardi ML Agouni A Heymes C Sargentini C. Circulating microparticles from patients with septic shock exert protective role in vascular function. Am J Respir Crit Care Med. (2008) 178:1148–55. 10.1164/rccm.200712-1835OC 18723433

[18] Priou P Gagnadoux F Tesse A Mastronardi ML Agouni A Meslier N. Endothelial dysfunction and circulating microparticles from patients with obstructive sleep apnea. Am J Pathol. (2010) 177:974–83. 10.2353/ajpath.2010.091252 20566740 PMC2913357

[19] Tual-Chalot S Leonetti D Andriantsitohaina R Martinez MC. Microvesicles: intercellular vectors of biological messages. Mol Interv. (2011) 11:88–94. 10.1124/mi.11.2.5 21540467

[20] Zaborowski MP Balaj L Breakefield XO Lai CP. Extracellular vesicles: composition, biological relevance, and methods of study. Bioscience. (2015) 65:783–97. 10.1093/biosci/biv084 26955082 PMC4776721

[21] Morel O Jesel L Freyssinet JM Toti F. Cellular mechanisms underlying the formation of circulating microparticles. Arterioscler Thromb Vasc Biol. (2011) 31:15–26. 10.1161/ATVBAHA.109.200956 21160064

[22] Sinning JM Jansen F Hammerstingl C Meier A Losch J Rohwer K. Circulating microparticles decrease after cardiac stress in patients with significant coronary artery stenosis. Clin Cardiol. (2016) 39:570–7. 10.1002/clc.22566 27410166 PMC6490784

[23] Lee YJ Jy W Horstman LL Janania J Reyes Y Kelley RE. Elevated platelet microparticles in transient ischemic attacks, lacunar infarcts, and multiinfarct dementias. Thromb Res. (1993) 72:295–304. 8303669 10.1016/0049-3848(93)90138-e

[24] Bennett JS Berger BW Billings PC. The structure and function of platelet integrins. J Thromb Haemost. (2009) 7(Suppl. 1):200–5. 10.1111/j.1538-7836.2009.03378.x 19630800

[25] Nolan JP Jones JC. Detection of platelet vesicles by flow cytometry. Platelets. (2017) 28:256–62. 10.1080/09537104.2017.1280602 28277059 PMC5415413

[26] Sinauridze EI Kireev DA Popenko NY Pichugin AV Panteleev MA Krymskaya OV. Platelet microparticle membranes have 50- to 100-fold higher specific procoagulant activity than activated platelets. Thromb Haemost. (2007) 97:425–34. 10.1160/TH06-06-0313 17334510

[27] Morel O Toti F Hugel B Bakouboula B Camoin-Jau L Dignat-George F. Procoagulant microparticles: disrupting the vascular homeostasis equation? Arterioscler Thromb Vasc Biol. (2006) 26:2594–604. 10.1161/01.ATV.0000246775.14471.26 16990554

[28] Owens AP III Mackman N. Microparticles in hemostasis and thrombosis. Circ Res. (2011) 108:1284–97. 10.1161/CIRCRESAHA.110.233056 21566224 PMC3144708

[29] Switonska M Slomka A Sinkiewicz W Zekanowska E. Tissue-factor-bearing microparticles (MPs-TF) in patients with acute ischaemic stroke: the influence of stroke treatment on MPs-TF generation. Eur J Neurol. (2015) 22:e328–99. 10.1111/ene.12591 25370815

[30] Tan X Shi J Fu Y Gao C Yang X Li J. Role of erythrocytes and platelets in the hypercoagulable status in polycythemia vera through phosphatidylserine exposure and microparticle generation. Thromb Haemost. (2013) 109:1025–32. 10.1160/TH12-11-0811 23571603

